# Phylogenetic diversity statistics for all clades in a phylogeny

**DOI:** 10.1093/bioinformatics/btad263

**Published:** 2023-06-30

**Authors:** Siddhant Grover, Alexey Markin, Tavis K Anderson, Oliver Eulenstein

**Affiliations:** Department of Computer Science, Iowa State University, Ames, IA 50010, United States; Virus and Prion Research Unit, National Animal Disease Center, USDA-ARS, Ames, IA 50010, United States; Virus and Prion Research Unit, National Animal Disease Center, USDA-ARS, Ames, IA 50010, United States; Department of Computer Science, Iowa State University, Ames, IA 50010, United States

## Abstract

The classic quantitative measure of phylogenetic diversity (PD) has been used to address problems in conservation biology, microbial ecology, and evolutionary biology. PD is the minimum total length of the branches in a phylogeny required to cover a specified set of taxa on the phylogeny. A general goal in the application of PD has been identifying a set of taxa of size *k* that maximize PD on a given phylogeny; this has been mirrored in active research to develop efficient algorithms for the problem. Other descriptive statistics, such as the minimum PD, average PD, and standard deviation of PD, can provide invaluable insight into the distribution of PD across a phylogeny (relative to a fixed value of *k*). However, there has been limited or no research on computing these statistics, especially when required for each clade in a phylogeny, enabling direct comparisons of PD between clades. We introduce efficient algorithms for computing PD and the associated descriptive statistics for a given phylogeny and each of its clades. In simulation studies, we demonstrate the ability of our algorithms to analyze large-scale phylogenies with applications in ecology and evolutionary biology. The software is available at https://github.com/flu-crew/PD_stats.

## 1 Introduction

Habitat loss, biological invasions, emerging infectious diseases, and climate change are altering global ecological systems. Extinctions have vastly outpaced speciation events over the past century (Barnosky et al. 2011, Ceballos et al. 2015), and novel pathogens are emerging at an increasing rate with two pandemics within the past 20 years (Gibb et al. 2020, Keusch et al. 2022). Quantifying biodiversity is consequential as it permits assessment of when and where significant biological changes are occurring. Traditional approaches to measuring changes in diversity patterns have taken the form of generating phylogenetic trees from genetic sequence data and then extracting measures of phylogenetic diversity (PD) from the inferred trees (Cadotte et al. 2010).

**Figure 1.**
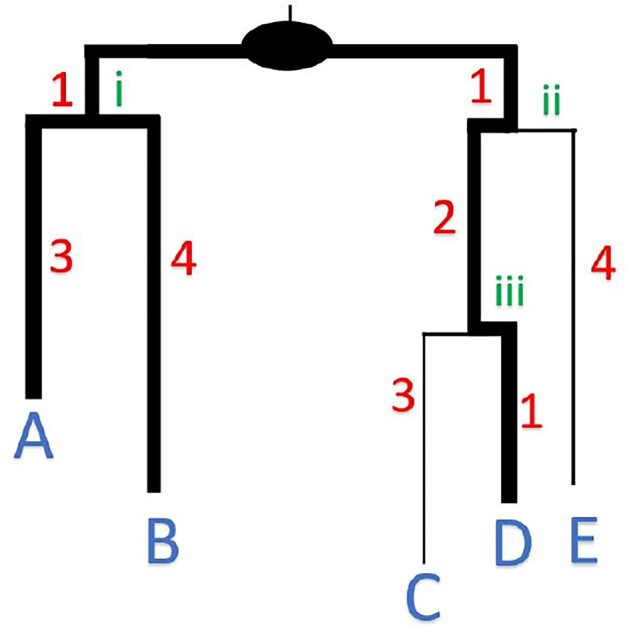
An example of a phylogenetic tree. The internal nodes below the root are labeled by roman numerals. The phylogenetic diversity of a set of taxa {*A*, *B*, *D*} is 3+4+1+1+2+1=12 as can be seen from the highlighted (bold) edges.

A now classic measure to quantify diversity is the PD index introduced by Faith (1992). PD measures the phylogenetic history among taxa occurring in a given sample and is calculated on a rooted phylogenetic tree for a set of taxa in the tree (i.e. represented as leaves). It is defined as the sum of the edge lengths that span these taxa along with the root of the tree (Fig. 1). This index directly interprets the evolutionary history of the taxon set and has been applied to generate solutions to practical problems in ecology and evolutionary biology (Li et al. 2020). Consequently, there has been extensive computational work considering the mathematical and combinatorial properties of the index (Steel 2005, Hartmann and Steel 2006, Minh et al. 2006, Moulton et al. 2007, Bordewich and Semple 2012). Specifically, there are polynomial time algorithms to solve the maximum PD problem on phylogenetic trees (Halldórsson et al. 1999, Steel, 2005, Minh et al. 2006, Hartmann and Steel 2006). However, PD applications (e.g. Faith and Richards 2012, Faith 2018, Faith and Simon 2018) could benefit from understanding descriptive statistics associated with PD across the entire phylogeny in addition to the maximum PD and the maximal PD set. These statistics allow for the identification of critical nodes within the phylogeny and for comparison of diversity statistics between nodes.

There has been progress toward calculating the minimum, average, and variance of PD on a phylogeny with *n* leaves and a subset of those leaves of size *k*. Hartmann et al. proposed using the minimum PD value to compute the gain in PD for the given set of taxa when compared with the worst-case scenario (i.e. a set of taxa with the smallest PD) (Hartmann and André 2013). Similarly, Manson et al. suggested using minimum PD to find the worst-case conservation scenario — i.e. find *k* taxa whose extinction will decrease the overall PD the most (Manson et al. 2022). Both Hartmann and André (2013) and Manson et al. (2022) provided algorithms for finding a value and a set for minimum PD. However, the complexity of the algorithm was not analyzed by Hartmann and André (2013), and the algorithm described by Manson et al. (2022) is prohibitive for larger phylogenies (and larger *k*) due to its $O(nk^{3})$ run time. The average PD was also explored by Hartmann et al. to compute how “beneficial” a taxon set is when compared with a randomly chosen set of *k* taxa (Hartmann and André 2013), i.e. to identify sets of taxa with higher than average PD. Tsirogiannis et al. (2012, 2014) proposed algorithms to compute both average PD and variance PD. Though these approaches computed the PD statistics *approximately* in linear time, they do not have an approximation guarantee as they approximate ratios of binomial coefficients using the hypergeometric distribution.

In this work, we introduce dynamic programming algorithms designed to efficiently and *accurately* compute the distribution of PD on a phylogeny by calculating the associated descriptive statistics for each clade on a given tree. Our solution for the Minimum PD problem has O(kn) time-complexity, significantly improving on the previous algorithm by Manson et al. (2022) that runs in $O(nk^{3})$ time, where *n* is the number of taxa of a phylogeny and *k* is the user-specified size of a PD set (query size). We achieved this complexity bound by applying the technique from Halldórsson et al. to our dynamic programming formulation (Tamir 1996, Halldórsson et al. 1999). We extended our dynamic programming framework to the Average PD and Variance PD problems, obtaining precise algorithms that run in $O(k^{2}n{\;\text{log}}^{2}n)$ time. We designed exact algorithms using big-number arithmetics with binomial coefficients. Crucially, our algorithms compute PD statistics (minimum, maximum, average, and variance) not only for the overall tree but also for every clade in the tree. Moreover, given a fixed input value *k*, the algorithms compute PD statistics for all $2\leq k_{i}\leq k$. Using a simulation study, we demonstrated that our dynamic programming framework can process large-scale trees with 10,000 taxa in under an hour on a standard PC, computing all of the PD statistics for each clade and every taxon size *k*, where 1<k<10 ,000.

## 2 Basics and preliminaries

We review basic definitions, statistical concepts, and terminology from computational phylogenetics that are needed in the context of this work. Once the PD is defined on a phylogenetic tree, we introduce the statistical measures of PD and finally formalize our problem of computing these statistics on all clades for a given phylogeny.

### 2.1 Basic definitions

A *(phylogenetic) tree* T:=(V(T),E(T),w), over a taxon set *X*, is a rooted full binary tree where each leaf is uniquely labeled with a taxon from *X* and edges are weighed by $w:E(T)\to R^{+}$. For convenience, we identify the taxa set with the leaf set, and *ρ* denotes the root of T.

A *subtree* induced by S⊆X is the minimal subtree of T connecting the vertices in S∪{ρ}; this subtree is denoted by *T_S_*. Further, the *subtree* rooted at v∈V(T) is denoted by *T_v_*, and the number of leaves in *T_v_* is denoted by |v|. *v^k^* is a collection of all subsets of *X* of size *k*, in the tree rooted at *v*, i.e. $v^{k}:=\{S|S\subseteq X\cap V(T_{v}),|S|=k\}.$

Let T be a phylogenetic tree, for a subset S⊆X, the *PD* of S, also referred to as *PD*, is


$$PD_{T}(S):=w(T_{S}).$$

For convenience, we write *PD*(*S*) when T is clear from the context.

We use $w(v^{↓})$ to denote the weight of the edge incident to *v* from the path between *ρ* and *v*. In addition, we define *w*(*T*) as the sum of the weights of all edges in T.

### 2.2 PD statistics and problems

We define the PD statistics and then introduce the corresponding computational problems.

To define the PD statistics let T be a phylogenetic tree over a taxon set of size n and k be an integer, where 1≤k≤n.

- The *minimum PD (MinPD)* is defined as
  $$\delta_{\left(k\right)}:=\underset{S\in \rho^{k}}{\min}PD(S).$$
  A *minimum PD set* is a set $S\in \rho^{k}$ where $PD(S)=\delta_{\left(k\right)}$.
- The *maximum PD (MaxPD)* is defined as
  $$\Delta_{\left(k\right)}:=\underset{S\in \rho^{k}}{\max}PD(S).$$
  A *maximum PD set S* is a set $S\in \rho^{k}$ where $PD(S)=\Delta_{\left(k\right)}$.
- The *average PD (AvgPD)* is defined as
  $$\alpha_{\left(k\right)}:=\frac{\sum_{S\in \rho^{k}}PD(S)}{\left(\begin{array}{c} n\\ k \end{array}\right)}.$$
- The *variance of PD (VarPD)* is defined as
  $$\psi_{\left(k\right)}:=\frac{\sum_{S\in \rho^{k}}(PD(S)-\alpha_{\left(k\right)}{)}^{2}}{\left(\begin{array}{c} n\\ k \end{array}\right)}.$$

The PD statistics can be computed at every clade of T. For instance, in Fig. 1, if we restrict the tree to the subtree at node *ii*, then one can compute that $\delta_{\left(2\right)}=6$ (S={C,D}), $\Delta_{\left(2\right)}=9$ (S={C,E}), $\alpha_{\left(2\right)}=7.33,$ and $\psi_{\left(2\right)}=1.55$.

Finally, we introduce the PD statistics problems that are based on the above-mentioned definitions.

#### 2.2.1 PD problems

Given a phylogenetic tree T over a taxon set of size n and an integer k, where 1≤k≤n, compute

- *MinPD Problem:* $\delta_{\left(k\right)}$ , and a Minimal PD set *S_min_*
- *MaxPD Problem:* $\Delta_{\left(k\right)}$ , and a Maximal PD set *S_max_*
- *AvgPD Problem:* $\alpha_{\left(k\right)}$

- *VarPD Problem:* $\psi_{\left(k\right)}$

The above statistics provide information about the distribution of PD on a fixed tree. We further extend the above problems to compute the aggregate statistics, not only for *T* overall but also for each *individual clade* in *T*. Note that for a clade at a node *v*, we only compute the above statistics for k≤|v| (for k>|v|, the corresponding values are undefined).

## 3 Solving PD Problems

We introduce efficient dynamic programing algorithms to solve the PD problems for all clades in a given tree with *n* leaves and an integer *k*. First, we prove a O(kn) recurrence for the minimum and maximum PD problems. Then, for the first time, we describe efficient algorithms for exactly solving the average PD and variance PD problems in $O(k^{2}n{\;\text{log}}^{2}n)$ time.

### 3.1 Minimum PD problem

We define δ(v,k)-subsolutions at subtrees and describe an O(kn) time algorithm to compute $\delta_{\left(k\right)}$ for each clade in *T* with taxon set *X*.

In a tree *T*, the subproblem at v∈V(T), for the *MinPD problem*, is defined as the problem to compute $\delta_{\left(k\right)}$ for *T_v_*. Thus, for 1≤k≤|v| in a tree rooted at a node *v*, we define δ(v,k) to be the $\min\;PD_{T_{v}}(S)$. That is, and $\delta (\rho ,k)=\delta_{\left(k\right)}$.

**Proposition 1***For every node* v∈V(T)*with children x and y (if they exist), and* 1≤k≤|v|*:*δ(v,k)=0*, if* v∈X*, elsewhere* $w(x^{↓)}$*is the weight of the edge from v to x, and* $w(y^{↓)}$*is the weight of the edge from v to y.*

**Proof.** For *v* being a leaf and any *k* (the base case), δ(v,k)=0, since there is no diversity to be selected from the subtree rooted at a leaf. For an internal node *v* with children *x* and *y*, there are multiple possibilities for splitting *k* taxa between the subtrees rooted at *x* and *y*.

$$\delta (v,k):=\underset{S\in v^{k}}{\min}PD_{T_{v}}(S),$$

$$\delta (v,k)=\min\{\begin{array}{c} w(x^{↓})+\delta (x,k),\\ w(y^{↓})+\delta (y,k),\\ w(x^{↓})+w(y^{↓})+\underset{\begin{array}{c} r+l=k\\ 1\leq r\leq |x|\\ 1\leq l\leq |y| \end{array}}{\min}(\delta (x,r)+\delta (y,l)) \end{array}$$

- *Case I*: all the *k* taxa in a minimal PD set *S* are from the subtree rooted at *x*. Note that for any $S\in x^{k}$, we have $PD_{T_{v}}(S)=PD_{T_{x}}(S)+w(x^{↓)}$. Therefore, set *S* that minimizes $PD_{T_{x}}(S)$ also minimizes $PD_{T_{v}}(S)$. That is,
  $$\delta (v,k)=\delta (x,k)+w(x^{↓)}.$$
- *Case II*: all the *k* taxa in a minimal PD set *S* are from the subtree rooted at *y*. Similarly to Case I, in this case
  $$\delta (v,k)=\delta (y,k)+w(y^{↓)}.$$
- *Case III*: *k* taxa are split between the subtrees rooted at *x* and *y*. Let us fix some *k*_1_ and *k*_2_, such that $1\leq k_{1}\leq |x|,\;1\leq k_{2}\leq |y|,\;k_{1}+k_{2}=k$. Then for $S_{1}\in x^{k_{1}}$ and $S_{2}\in y^{k_{2}}$, we have
  $$PD_{T_{v}}(S_{1}\cup S_{2})=PD_{T_{x}}(S_{1})+PD_{T_{y}}(S_{2})+w(x^{↓)}+w(y^{↓}).$$

Then minimizing over all such sets $S_{1}$ and $S_{2}$ reduces to independent minimization in the left and right subtrees. It is then not difficult to see that


$$\begin{array}{c} \underset{S\in v^{k}}{\min}PD(S)=w(x^{↓})+w(y^{↓})+\underset{\begin{array}{c} k_{1}+k_{2}=k\\ 1\leq k_{1}\leq |x|\\ 1\leq k_{2}\leq |y| \end{array}}{\min}(\delta (x,k_{1})+\delta (y,k_{2})). \end{array}$$

Combining all three above cases, we obtain the formula from Proposition 1. □

Our pseudocode for the algorithm using the recurrence above is presented in Algorithm 1.

Algorithm 1 MinPD Problem

1: **procedure**ComputeMin(*T*, *k*)

2: δ←[1;2n−1][0;k]

3:    **for**v∈X**do**

4:      **for** *i *=* *0 to *k* **do**

5:        δ[v,i]=0

6:      **end for**

7:    **end for**

8:    **for**v∈V∖X(in post order) **do**

9:     //*x*, *y*are children of*v*

10:      **for** *i *=* *1 to min(k,|v|)**do**

11:        minval=∞

12:        **for**r=max(0,i−|y|) to min(|x|,i)**do**

13:          l←i−r

14:          val = $\delta [x,r]+w(x^{↓})\cdot \min(r,1)$

15:           + $\delta [y,l]+w(y^{↓})\cdot \min(l,1)$

16:          **if**val < minval

17:           minval = val

18:      **end for**

19:      δ[v,k]=val

20:     **end for**

21:    **end for**

22: **end procedure**

**Theorem 2** *Algorithm 1 solves the MinPD problem in O(kn) time.*

**Proof.** Algorithm 1 is built on the recurrence relation proved in Proposition 1. The complexity of Algorithm 1 is as follows.

Line 2 creates an empty 2D array of O(kn) space. The outer loop in line 3 runs in *O*(*n*) time, while the inner loop in line 4 runs in *O*(*k*) time; thus the runtime for lines 3−7 is O(kn). At first, it is clear that lines 8−20 take $O(k^{2}n)$ time; however, Tamir (1996) and Halldórsson et al. (1999) proved that dynamic programming of this type in fact runs in O(kn) time. We explicitly summarize their results in Lemma 3.

**Lemma 3** (Tamir 1996, Halldórsson et al. 1999) *For a non-leaf node v with children x and y, let I_v_ denote the number of pairs (l, r) such that* 0≤l≤|x|, 0≤r≤|y|*, and* l+r≤min(k,|v|)*. Then*,

$$I(T):=\sum_{v\in V(T)}I_{v}\in O(kn)$$

It is not difficult to see that *I*(*T*) bounds the runtime of Algorithm 1.

Thus, MinPD for every clade can be computed in *O*(*kn*) time and space. Once $\delta_{\left(k\right)}$ is obtained, the Minimal PD set can be found via backtracking in O(k+n) time. □

### 3.2 Maximum PD problem

The original MaxPD problem can be solved in *O*(*n*) time (Spillner et al. 2008). Then, solving this problem for every clade in a tree would require $O(n^{2})$ time. However, adapting the dynamic programming approach from the previous section, we can solve the same problem in O(kn) time, which is more desirable, especially for smaller *k*. The adaptation would only require changing the minimization criterion in Algorithm 1 to maximization. Additionally, it might be possible to extend the algorithm from Spillner et al. (2008) to achieve a similar runtime.

### 3.3 Average PD problem

We begin by defining AvgPD subproblems and then prove the recurrence relation for computing the β(v,k)-subsolution at *T_v_* (defined below). We then present an $O(k^{2}n{\;\text{log}}^{2}n)$ time and $O(k^{2}n\;\text{log}\;n)$ space algorithm to compute $\alpha_{\left(k\right)}$ for each clade in *T*. We define $\beta_{\left(k\right)}:=\sum_{S\in \rho^{k}}PD(S)$. That is, $\beta_{\left(k\right)}=\alpha_{\left(k\right)}\cdot \left(\begin{array}{c} \left|X\right|\\ k \end{array}\right)$. We then define the subproblem β(v,k) at a vertex v∈V(T), for the AvgPD problem, as the problem to compute $\beta_{\left(k\right)}$ in *T_v_* subtree. That is, for 1≤k≤|v|,


$$\beta (v,k):=\sum_{S\in v^{k}}PD_{T_{v}}(S).$$

Then, $\beta_{\left(k\right)}=\beta (\rho ,k)$ and $\alpha_{\left(k\right)}=\beta_{\left(k\right)}/\left(\begin{array}{c} \left|X\right|\\ k \end{array}\right)$.

**Proposition 4** *For every node* v∈V(T)*with children x and y (if they exist), and* 1≤k≤|v|*:*

β(v,k)=0, *if* v∈X*, else* β(v,k)=

$$\begin{array}{cc} & \left[\underset{\begin{array}{c} r+l=k\\ 1\leq r\leq |x|\\ 1\leq l\leq |y| \end{array}}{\sum }\left(\begin{array}{c} \left|y\right|\\ l \end{array}\right)\beta (x,r)+\left(\begin{array}{c} \left|x\right|\\ r \end{array}\right)\beta (y,l\right)\\ & +\left(\begin{array}{c} \left|x\right|\\ r \end{array}\right)\left(\begin{array}{c} \left|y\right|\\ l \end{array}\right)\left(w(x^{↓})+w(y^{↓})\right)]\\ & +[\beta (x,k)+\left(\begin{array}{c} \left|x\right|\\ k \end{array}\right)w(x^{↓})]+[\beta (y,k)+\left(\begin{array}{c} \left|y\right|\\ k \end{array}\right)w(y^{↓})] \end{array}$$

**Proof.** For $v\in X,\;\beta (v,1)=PD_{T_{v}}(\{v\})=0$. We now prove the recurrence for internal *v* with children *x* and *y*. We split $\beta (v,k)=\sum_{S\in v^{k}}PD_{T_{v}}(S)$ into three categories.

- *Case I* : all the k taxa in *S* are chosen from the subtree rooted at *x*, i.e. $S\in x^{k}$. Then,
  $$\beta_{I}(v,k)=\sum_{S\in x^{k}}(PD(S)+w(x^{↓}))=\beta (x,k)+\left(\begin{array}{c} \left|x\right|\\ k \end{array}\right)w(x^{↓}).$$
- *Case II* : all the k taxa in *S* are chosen from the subtree rooted at *y*. Similarly to Case I, we have
  $$\beta_{II}(v,k)=\beta (y,k)+\left(\begin{array}{c} \left|y\right|\\ k \end{array}\right)w(y^{↓}).$$
- *Case III* : *k* taxa are split between subtrees rooted at *x* and *y*. Let *k*_1_ and *k*_2_ be such that $1\leq k_{1}\leq |x|,\;1\leq k_{2}\leq |y|,\;k_{1}+k_{2}=k$. Then we define
  $$\beta (v,k_{1},k_{2}):=\sum_{\begin{array}{c} S_{1}\in x^{k_{1}}\\ S_{2}\in y^{k_{2}} \end{array}}PD_{T_{v}}(S_{1}\cup S_{2}).$$

It is then not difficult to see that


$$\beta_{\mathrm{III}}(v,k)=\underset{\begin{array}{c} k_{1}+k_{2}=k\\ 1\leq k_{1}\leq |x|\\ 1\leq k_{2}\leq |y| \end{array}}{\sum }\beta (v,k_{1},k_{2}).$$

Next, observe the following

$$\begin{array}{c} \beta (v,k_{1},k_{2})=\\ \sum_{\begin{array}{c} S_{1}\in x^{k_{1}}\\ S_{2}\in y^{k_{2}} \end{array}}(PD_{T_{x}}(S_{1})+PD_{T_{y}}(S_{2})+w(x^{↓})+w(y^{↓}))\\ =\sum_{S_{1}\in x^{k_{1}}}(\left(\begin{array}{c} \left|y\right|\\ k_{2} \end{array}\right)(PD_{T_{x}}(S_{1})+w(x^{↓})+w(y^{↓}))+\beta (y,k_{2}))\\ =\left(\begin{array}{c} \left|x\right|\\ k_{1} \end{array}\right)\left(\begin{array}{c} \left|y\right|\\ k_{2} \end{array}\right)(w(x^{↓})+w(y^{↓})))+\left(\begin{array}{c} \left|y\right|\\ k_{2} \end{array}\right)\beta (x,k_{1})+\left(\begin{array}{c} \left|x\right|\\ k_{1} \end{array}\right)\beta (y,k_{2}) \end{array}$$

Then, observing gives us the equation in Proposition 4. □


$$\beta (v,k)=\beta_{I}(v,k)+\beta_{II}(v,k)+\beta_{\mathrm{III}}(v,k)$$

Finally, we have where α(v,k) is the solution for the the problem to compute $\alpha_{\left(k\right)}$ in *T_v_*. Our pseudo code for the algorithm using the recurrence above is presented above.


$$\alpha (v,k)=({\left(\begin{array}{c} \left|v\right|\\ k \end{array}\right)}^{-1})\cdot \beta (v,k)$$

**Algorithm 2** AvgPD Algorithm

1: **procedure**ComputeAvg(*T*, *k*)

2: Precompute $\left(\begin{array}{c} i\\ j \end{array}\right)\;\text{values for}\;i\leq n,j\leq k$ via a Pascal triangle.

3:   α←[1;2n−1][1;k]

4:   β←[1;2n−1][1;k]

5:   **for**v∈X**do**

6:     α[v,1]=0

7:     β[v,1]=0

8:   **end for**

9:   **for**v∈V∖X(in post order) **do**

10:    //*x*, *y*are children of*v*

11:     **for** *i *=* *1 to min(k,|v|)**do**

12:       sum_x = $\beta [x,i]+\left(\begin{array}{c} \left|x\right|\\ i \end{array}\right)w(x^{↓})$

13:       sum_y = $\beta [y,i]+\left(\begin{array}{c} \left|y\right|\\ i \end{array}\right)w(y^{↓})$

14:       sum = sum_x + sum_y

15:       **for**r=max(1,i−|y|) to min(|x|,i−1)**do**

16:         l←i−r

17:         $\text{sum}+=\left(\begin{array}{c} \left|y\right|\\ l \end{array}\right)\beta [x,r]+\left(\begin{array}{c} \left|x\right|\\ r \end{array}\right)\beta [y,l]$

18:            $+\left(\begin{array}{c} \left|x\right|\\ r \end{array}\right)\left(\begin{array}{c} \left|y\right|\\ l \end{array}\right)(w(x^{↓)}+w(y^{↓}))$

19:       **end for**

20:       β[v,i]=sum

21:       α[v,i]=sum$/\left(\begin{array}{c} \left|v\right|\\ i \end{array}\right)$

22:     **end for**

23:    **end for**

24: **end procedure**

**Theorem 5** *Algorithm 2 solves the AvgPD in* $O(k^{2}n{\;\text{log}\;}^{2}n)$*time and* $O(k^{2}n\;\text{log}\;n)$*space.*

**Proof.** The analysis of the complexity of Algorithm 2 is similar to the analysis of Algorithm 1. The main difference is the fact that $\left(\begin{array}{c} n\\ k \end{array}\right)$ values can be very large, hence we need to account for the arithmetic on large numbers. Note that $\left(\begin{array}{c} n\\ k \end{array}\right)\leq n^{k}$; consequently, the number of bits required to encode such numbers is $O(\text{log}(n^{k}))=O(k\;\text{log}\;n)$. Note that precomputing all values $\left(\begin{array}{c} i\\ j \end{array}\right)$ for i≤n and j≤k can be done by computing a part of a Pascal triangle in O(kn·k log n) time. The remainder of the algorithm requires big-number arithmetic. Note that multiplication of two O(k log n)-bit numbers can be done in $O(k\;\text{log}n\;\text{log}\;k)=O(k{\;\text{log}\;}^{2}n)$ time (Harvey and Van Der Hoeven 2021). Therefore, the remainder of the algorithm requires $O(kn\cdot k{\;\text{log}\;}^{2}n)$ time, where *kn* follows from the proof of Theorem 2. □

### 3.4 Variance PD problem

Recall that by definition,


$$\begin{array}{c} \psi_{\left(k\right)}:=\frac{\sum_{S\in \rho^{k}}(PD(S)-\alpha_{\left(k\right)}{)}^{2}}{\left(\begin{array}{c} \left|X\right|\\ k \end{array}\right)}\\ =({\left(\begin{array}{c} \left|X\right|\\ k \end{array}\right)}^{-1})\cdot (\sum_{S\in \rho^{k}}PD{\left(S\right)}^{2}+\sum_{S\in \rho^{k}}\alpha_{\left(k\right)}^{2}-2\sum_{S\in \rho^{k}}PD(S)\cdot \alpha_{\left(k\right)})\\ =\frac{\sum_{S\in \rho^{k}}PD{\left(S\right)}^{2}+\left(\begin{array}{c} \left|X\right|\\ k \end{array}\right)\alpha_{\left(k\right)}^{2}-2\left(\begin{array}{c} \left|X\right|\\ k \end{array}\right)\alpha_{\left(k\right)}^{2}}{\left(\begin{array}{c} \left|X\right|\\ k \end{array}\right)}\\ =\frac{\sum_{S\in \rho^{k}}PD{\left(S\right)}^{2}}{\left(\begin{array}{c} \left|X\right|\\ k \end{array}\right)}-\alpha_{\left(k\right)}^{2} \end{array}$$

Using Algorithm 2, we can compute $\alpha_{\left(k\right)}$ in $O(k^{2}n\;\text{log}\;n)$ time. Therefore, we are only left to compute the sum of squares $\sum_{S\in \rho^{k}}PD{\left(S\right)}^{2}$, which we denote by $\gamma_{\left(k\right)}$. For convenience, we also define for any v∈V(T) and 1≤k≤|v|.


$$\gamma (v,k):=\sum_{S\in v^{k}}PD_{T_{v}}{\left(S\right)}^{2},$$

**Proposition 6** *For every node* v∈V(T)*with children x and y (if exist), and* 1≤k≤|v|,

γ(v,k)=0, *if* v∈X*, else*

$$\begin{array}{c} \gamma (v,k)=\sum_{\begin{array}{c} 1\leq r,l\leq k-1\\ r+l=k \end{array}}[\left(\begin{array}{c} \left|y\right|\\ l \end{array}\right)\gamma (x,r)+\left(\begin{array}{c} \left|x\right|\\ r \end{array}\right)\gamma (y,l)+\left(\begin{array}{c} \left|x\right|\\ r \end{array}\right)\left(\begin{array}{c} \left|y\right|\\ l \end{array}\right)\lambda^{2}\\ +2(\beta (x,r)\beta (y,l)+\lambda (\left(\begin{array}{c} \left|y\right|\\ l \end{array}\right)\beta (x,r)+\left(\begin{array}{c} \left|x\right|\\ r \end{array}\right)\beta (y,l)))]\\ +[\gamma (x,k)+\left(\begin{array}{c} \left|x\right|\\ k \end{array}\right)w(x^{↓}){}^{2}+2w(x^{↓})\beta (x,k)]\\ +[\gamma (y,k)+\left(\begin{array}{c} \left|y\right|\\ k \end{array}\right)w(y^{↓}){}^{2}+2w(y^{↓})\beta (y,k)] \end{array}$$

*where λ represents* $w(x^{↓})+w(y^{↓})$*and* $\beta (v,k)=\sum_{S\in v^{k}}PD_{T_{v}}(S)$.

**Proof.** For a leaf $v\in X,\;\gamma (v,1)=PD_{T_{v}}{\left(\{v\}\right)}^{2}=0$. We now prove the recurrence for an internal vertex *v* with children *x* and *y*. We split γ(v,k) into three categories.

- *Case I*: all the k taxa in *S* are chosen from the subtree rooted at *x*, i.e. $S\in x^{k}$. Then,
  $$\begin{array}{c} \gamma_{I}(v,k)=\sum_{S\in x^{k}}(PD_{T_{x}}(S)+w(x^{↓}){)}^{2}\\ =\gamma (x,k)+\left(\begin{array}{c} \left|x\right|\\ k \end{array}\right)w{\left(x^{↓}\right)}^{2}+2w(x^{↓})\beta (x,k). \end{array}$$
- *Case II*: all the k taxa in *S* are chosen from the subtree rooted at *y*. Similarly to Case I, we have
  $$\gamma_{II}(v,k)=\gamma (y,k)+\left(\begin{array}{c} \left|y\right|\\ k \end{array}\right)w(y^{↓}){}^{2}+2w(y^{↓})\beta (y,k),$$
- *Case III* : *k* taxa are split between the subtrees rooted at *x* and *y*. Let *k*_1_ and *k*_2_ be such that $1\leq k_{1}\leq |x|,\;1\leq k_{2}\leq |y|,\;k_{1}+k_{2}=k$. For convenience, we assign $\lambda :=w(x^{↓})+w(y^{↓})$. Then, we define
  $$\gamma (v,k_{1},k_{2}):=\sum_{\begin{array}{c} S_{1}\in x^{k_{1}}\\ S_{2}\in y^{k_{2}} \end{array}}PD_{T_{v}}{\left(S_{1}\cup S_{2}\right)}^{2}$$

Observe now that


$$\begin{array}{c} \gamma (v,k_{1},k_{2})=\sum_{\begin{array}{c} S_{1}\in x^{k_{1}}\\ S_{2}\in y^{k_{2}} \end{array}}{\left(PD_{T_{x}}(S_{1})+PD_{T_{y}}(S_{2})+\lambda \right)}^{2}\\ =\sum_{\begin{array}{c} S_{1}\in x^{k_{1}}\\ S_{2}\in y^{k_{2}} \end{array}}(PD_{T_{x}}{\left(S_{1}\right)}^{2}+PD_{T_{y}}{\left(S_{2}\right)}^{2}+\lambda^{2}+\\ +2\lambda (PD_{T_{x}}(S_{1})+PD_{T_{y}}(S_{2}))+\\ +2PD_{T_{x}}(S_{1})PD_{T_{y}}(S_{2}))\\ =\left(\begin{array}{c} \left|y\right|\\ k_{2} \end{array}\right)\gamma (x,k_{1})+\left(\begin{array}{c} \left|x\right|\\ k_{1} \end{array}\right)\gamma (y,k_{2})+\left(\begin{array}{c} \left|x\right|\\ k_{1} \end{array}\right)\left(\begin{array}{c} \left|y\right|\\ k_{2} \end{array}\right)\lambda^{2}\\ +2\lambda (\left(\begin{array}{c} \left|y\right|\\ k_{2} \end{array}\right)\beta (x,k_{1})+\left(\begin{array}{c} \left|x\right|\\ k_{1} \end{array}\right)\beta (y,k_{2}))\\ +2\beta (x,k_{1})\beta (y,k_{2}) \end{array}$$

and $\gamma_{\mathrm{III}}(v,k)=\underset{\begin{array}{c} k_{1}+k_{2}=k\\ 1\leq k_{1}\leq |x|\\ 1\leq k_{2}\leq |y| \end{array}}{\sum }\gamma (v,k_{1},k_{2})$.

Proposition 7 then follows from the observation that

$$\gamma (v,k)=\gamma_{I}(v,k)+\gamma_{II}(v,k)+\gamma_{\mathrm{III}}(v,k).$$

□

**Theorem 7** *Algorithm 3 solves the VarPD in in* $O(k^{2}n{\;\text{log}\;}^{2}n)$*time and* $O(k^{2}n\;\text{log}\;n)$*space.*

**Proof.** The proof is similar to the proof of Theorem 5. □

Algorithm 3 VarPD Algorithm

1: **procedure**ComputeVarPD(*T*, *k*)

2:   Compute $\left(\begin{array}{c} i\\ j \end{array}\right)\;\text{values for}\;i\leq n,j\leq k$ via a Pascal triangle.

3:   Compute *α* and *β* arrays using Algorithm 2.

4:   ψ←[1;2n−1][1;k]; γ←[1;2n−1][1;k]

5:   **for**v∈X**do**

6:     **for** *i *=* *1 to *k* **do**

7:       ψ[v,i]=γ[v,i]=0

8:     **end for**

9:   **end for**

10:   **for**v∈V∖X (in post order) **do**

11:    //*x*, *y*are children of*v*

12:     **for** *i *=* *1 to min(k,|v|)**do**

13:   sq_x= $\gamma (x,k)+\left(\begin{array}{c} \left|x\right|\\ k \end{array}\right)w{\left(x^{↓}\right)}^{2}+2w(x^{↓})\beta [x,k]$

14:   sq_y= $\gamma (y,k)+\left(\begin{array}{c} \left|y\right|\\ k \end{array}\right)w(y^{↓}){}^{2}+2w(y^{↓})\beta [y,k]$

15:   sq = sq_x + sq_y

16:   **for**r=max(0,i−|y|) to min(|x|,i)**do**

17:         sq += $\left(\begin{array}{c} \left|y\right|\\ l \end{array}\right)\gamma [x,r]+\left(\begin{array}{c} \left|x\right|\\ r \end{array}\right)\gamma [y,l]$

18:         $+\left(\begin{array}{c} \left|x\right|\\ r \end{array}\right)\left(\begin{array}{c} \left|y\right|\\ l \end{array}\right)\lambda^{2}+2[\beta [x,r]\beta [y,l]$

19:         $+\lambda (\left(\begin{array}{c} \left|y\right|\\ l \end{array}\right)\beta [x,r]+\left(\begin{array}{c} \left|x\right|\\ r \end{array}\right)\beta [y,l])]$

20:   **end for**

21:       γ[v,i]=sq; $\psi [v,i]=\frac{\gamma [v,i]}{\left(\begin{array}{c} \left|v\right|\\ i \end{array}\right)}$-$\alpha {\left[v,i\right]}^{2}$

22:     **end for**

23:   **end for**

24: **end procedure**

## 4 Experimental evaluation

We demonstrate the applicability of the PD statistics algorithms by evaluating their scalability. Additionally, we compare our exact algorithms for calculating AvgPD and VarPD to calculating these measures through randomly sampling taxon sets. The algorithms were implemented in Python 3 and executed on a PC with an Intel Core i7 3.19 Ghz processor using 8.0 GB of RAM under the Windows 10 operating system. Dendropy (Sukamaran and Holder 2010) and Biopython (Cock et al. 2009) were used in our implementation.

### 4.1 Scalability analysis

**Figure 2.**
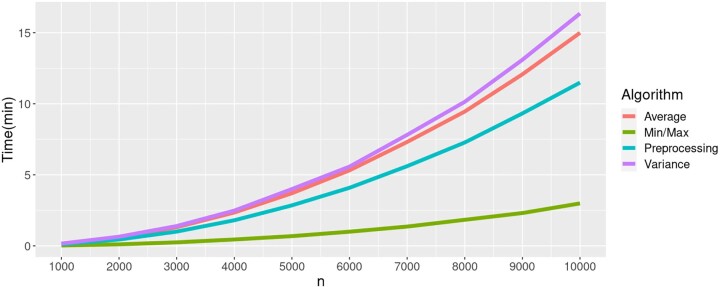
Runtime of the algorithms to compute PD statistics on trees with 1000 – 10,000 taxa for *k *=* n*. Since our algorithms for AvgPD and VarPD problems depend on the preprocessing step for computing the binomial coefficients, we report the runtime of those algorithms separately from the preprocessing runtime.

To demonstrate the applicability of our algorithms, we generated large-scale phylogenetic trees using the birth/death model (1.0 birth rate and 0.5 death rate) with the number *n* of taxa ranging from 1000 to 10,000 and a step of 1000. For each fixed number of taxa, we generated 10 trees. The PD set size *k* was set to match the total number of taxa *n* to measure the worst-case complexity of our algorithms. Thus, we compute the PD statistics for all clades in a tree and all *k* ranging from 2 to n−1. Figure 2 demonstrates that for a 10,000 taxon tree, all of the PD statistics on all clades (and all *k*) can be computed within an hour.

### 4.2 AvgPD and VarPD algorithms are significantly faster than random sampling

When exact algorithms for computing the mean/variance of a distribution are not known, random sampling is typically used to approximate those moments. Here we compare the runtime of our methods for AvgPD and VarPD problems against a random sampling strategy.

We simulated a birth/death tree (1.0 birth rate and 0.5 death rate) with *n *=* *500 taxa and tested different *k* between 50 and 450 with a step of 50. After computing the exact AvgPD and VarPD values using our algorithms, we sampled subsets of taxa of size *k* uniformly at random and computed the PD of the chosen samples. After every 200 samples, we computed the overall mean/variance PD of the samples until that value converged to the truth within 0.1%. Below, we report the average runtimes of the algorithms over various *k*.

For the AvgPD problem, random sampling required 14.2 s to converge, while our algorithm required 2 s to compute, on average. The difference was more significant for the VarPD problem, where random sampling required 28 min to converge (on average), while our algorithm required only 2.8 s. Further, for AvgPD, the gap between our algorithm and random sampling increases with larger *n*. For example, for *n *=* *2000 and *k *=* *20, random sampling required >1 h to converge, while our algorithm required only 6.5 s to complete.

## 5 Conclusion

In this work, we devised effective, efficient algorithms for computing the PD statistics. Notably, our algorithms are not restricted to calculating these values for the entire tree; but for every subtree/clade present in *T*. Furthermore, our algorithms provide descriptive statistics of PD, including the minimum PD, maximum PD, average PD, and variance PD for all values ranging from 1 up to *k*. A major merit of our algorithms for the minimum and maximum PD is that these values can be computed simultaneously by building on a similar dynamic programming approach. All the algorithms described in this work are efficient and scale well in practice. Consequently, these algorithms can be applied in the field without limitation based on the number of taxa or *k*.

Quantifying statistics such as the average PD and variance PD across all nodes in a phylogeny can provide deep insights into evolutionary histories. The *average PD* is the mean PD value over all sets of *k* taxa on a tree. In other words, it is the expected PD value if taxa were chosen uniformly at random. Similarly, the *variance PD* is the variance of the PD values given an equiprobable distribution of selecting any *k* taxa. Standardizing these diversity measures is crucial, making values comparable for different values of *k* and across different phylogenies. Further, when computed for all taxa in a phylogeny, these statistics can be used to assess the role of evolutionary history in shaping ecological communities, to determine how this evolutionary history influences niche and resource use, and can be used to study trait evolution and biogeography (Webb et al. 2002).

## Data Availability

The data underlying this article are available in P D_stats, at https://github.com/flucrew/PD_stats/.
